# RUNX1 promotes mitophagy and alleviates pulmonary inflammation during acute lung injury

**DOI:** 10.1038/s41392-023-01520-6

**Published:** 2023-08-07

**Authors:** Xiaoju Tang, Lichun Zhong, Xin Tian, Ying Zou, Silu Hu, Jia Liu, Ping Li, Min Zhu, Fengming Luo, Huajing Wan

**Affiliations:** 1 Department of Respiratory and Critical Care Medicine, Clinical Research Center for Respiratory Diseases, West China Hospital, Sichuan University, No. 37 Guo Xue Xiang, 610041 Chengdu, China; 2 Laboratory of Pulmonary Immunology and Inflammation, Frontiers Science Center for Disease-related Molecular Network, Sichuan University, No. 2222 Xin Chuan Road, 610200 Chengdu, Sichuan China

**Keywords:** Respiratory tract diseases, Inflammation

**Dear Editor**,

Acute lung injury (ALI) is a significant contributor to the development of acute respiratory distress syndrome (ARDS), which is a severe clinical condition associated with high morbidity and mortality.^1^ It is increasingly evident that preserving mitochondrial health in alveolar epithelial cells holds great therapeutic potential for ARDS.^2^ Mitophagy, a cellular process aimed at maintaining mitochondrial health, plays a critical role in this regard.^3^ Therefore, gaining a comprehensive understanding of the factors that regulate mitophagy in alveolar epithelial cells during ALI could greatly inform the development of future therapeutic approaches for ARDS.

In this study, we performed RNAseq analysis on RUNX1 silenced alveolar epithelial cells (A549) and found that expression of genes involved in the mitophagy pathway was significantly affected in RUNX1 silenced A549 cells (Fig. 1a). Among the differentially expressed genes (DEGs) associated with the mitophagy pathway (Table S1), six mitophagy adaptor proteins were consistently decreased (Supplementary Fig. S1a). Changes in the expression of mitophagy adaptor proteins (fold changes > 1.2) were further confirmed by RT-qPCR, revealing significant downregulation of mRNA levels of *P62*, *BNIP3*, and *BNIP3L* following RUNX1 silencing (Fig. 1b). These results suggest that RUNX1 regulates the activation of mitophagy by up-regulation of mitophagy adaptor proteins.

**Fig. 1:**
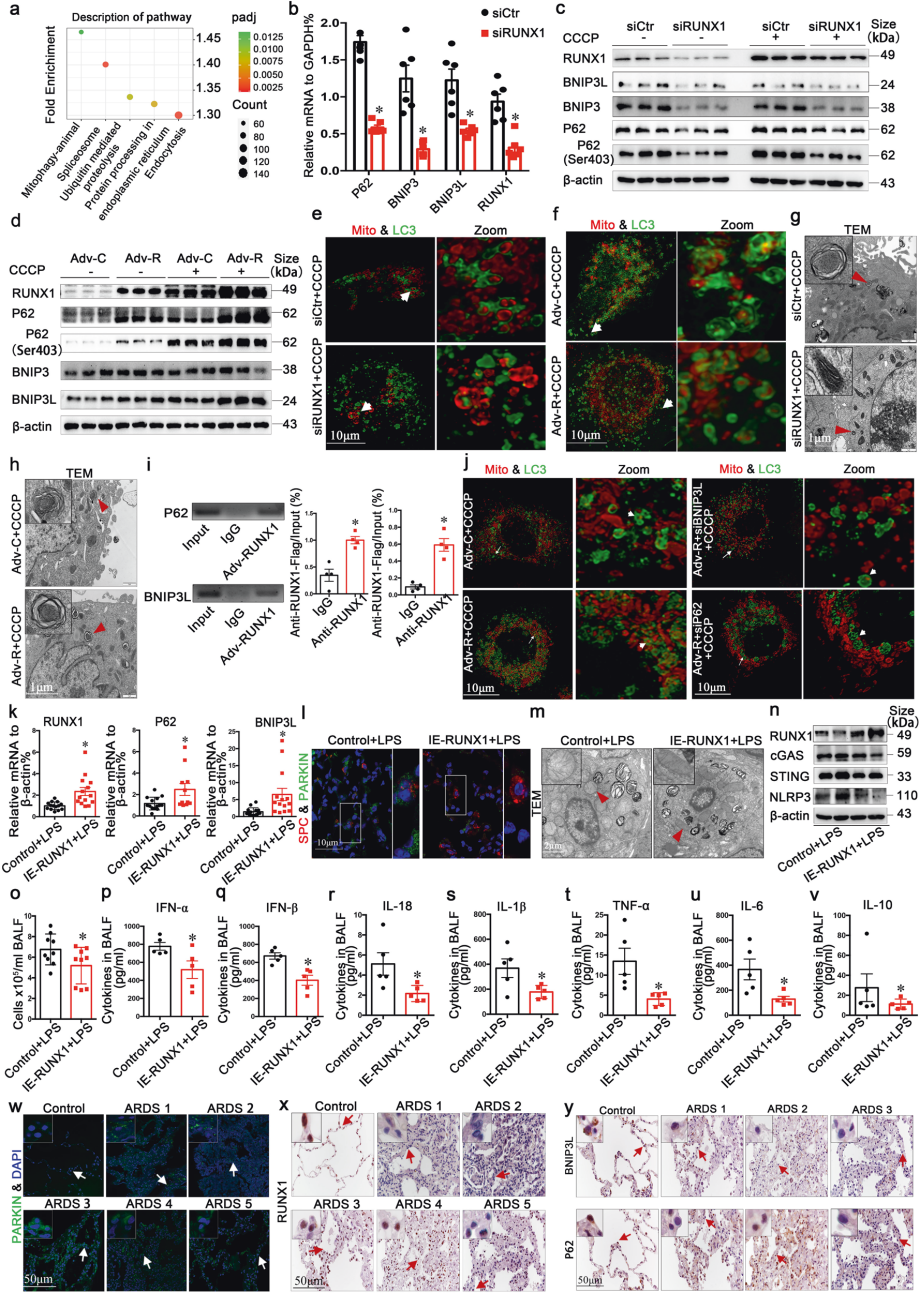
RUNX1 promotes mitophagy and alleviates pulmonary inflammation during acute lung injury**. a** Kyoto Encyclopedia of Genes and Genomes (KEGG) analysis of 7958 differentially expressed genes in RUNX1 silenced A549 cells was performed, and the top 5 significantly affected pathways were shown, including mitophagy. **b** Real-time PCR (RT-qPCR) verification of decreased expression of *P62*, *BNIP3*, and *BNIP3L* in RUNX1 silenced A549 cells. Data are represented as mean ± SEM, *n* = 6 per group. **c** and **d** Western blot of RUNX1, BNIP3, BNIP3L, P62, and phosphor-P62 (Ser403). Data are represented as mean ± SEM, *n* = 3 per group. **e** and **f** Immunofluorescence imaging of autophagosomes(green) and mitochondria(red) in A549 cells. Higher magnifications of the corresponding regions indicated by the arrows are shown in the right column. Arrowheads indicate damaged mitochondria (red) engulfed by an autophagosome (green). Scale bars: 10 μm. **g** and **h** Representative ultrastructural images show membrane-engulfed mitochondria in A549 cells. Insets are higher magnifications of the corresponding regions indicated by the red arrowheads. Scale bars: 1 μm. **i** Chromatin immunoprecipitation (ChIP)-PCR assays using *P62* and *BNIP3L* promoter-specific primers designed around the predicted RUNX1 binding sites. Data are represented as mean ± SEM, *n* = 3 per group. **j** Immunofluorescent imaging of autophagosomes (green) and mitochondria(red) in P62 and BNIP3L rescue experiments. Higher magnifications of the corresponding regions, indicated by the arrows are shown in the right column. Scale bars: 10 μm. **k** Real-time PCR analysis of *RUNX1*, *P62* and *BNIP3L* mRNAs in lung tissues of LPS-induced IE-RUNX1 mice. Data are represented as mean ± SEM, *n* = 14 per group. **l** Immunofluorescence co-staining of pro-SPC and PARKIN in the lungs 24 h after LPS administration. Higher magnifications of the boxed regions are shown on the right side of the images. Scale bars:10 μm. **m** Representative ultrastructure images show the morphological difference of mitochondria in AT2 cells between control and IE-RUNX1 mice 24 h after LPS administration. Arrows indicate the mitochondria. Scale bars: 2 μm. **n** Western blot for cGAS, STING, NLRP3, and β-actin using lung lysates from control and IE-RUNX1 mice. **o**–**v** ELISA analysis for IFN-α, IFN-β, IL-1β, IL-18, TNF-α, IL-6, and IL-10 in BALF collected from control and IE-RUNX1 mice. Data are represented as mean ± SEM, *n* = 5 per group. **w**–**y** Immunostaining of PARKIN, RUNX1, P62, and BNIP3L on the lung sections collected from ARDS patients and controls. Scale bars: 50 μm. *P < 0.05

To test whether RUNX1 is required for injury-induced mitophagy, A549 cells were treated with CCCP (20 μM) to induce mitophagy (Supplementary Fig. S1b, c). Silencing RUNX1 significantly inhibited the expression of P62, BNIP3, and BNIP3L (Fig. 1c and Supplementary Fig. S1d–h). Induced expression of RUNX1 significantly increased the expression of P62 and BNIP3L, but not BNIP3 (Fig. 1d and Supplementary Fig. S1i–m). To test whether RUNX1 was required for mitophagy adaptor proteins mediated engulfment of damaged mitochondria by autophagosome, A549 cells were co-transfected with Adv-HBmTur-Mito and Adv-EGFP-LC3 to label mitochondria and autophagosomes, respectively. Compared with controls, the number of the mitochondria surrounded by LC3 positive autophagosomes was significantly decreased in RUNX1 silenced A549 cells (Fig. 1e and Supplementary Fig. S1n) and was significantly increased in RUNX1 overexpressing A549 cells (Fig. 1f and Supplementary Fig. S1o). Ultramicroscopic observations demonstrated that membrane-engulfed mitochondria were readily detected in A549 cells after CCCP treatment, but not in RUNX1 silenced A549 cells (Fig. 1g). Moreover, more membrane-engulfed mitochondria were present in RUNX1-overexpressing A549 cells (Fig. 1h). Consistent with these, we observed that inhibition of RUNX1 disrupted the autophagic influx (Supplementary Fig. S1p, r) and overexpressing of RUNX1 promoted the autophagic influx (Supplementary Fig. S1q, s). These data demonstrate that RUNX1 is required to promote mitophagy in A549 cells after CCCP treatment.

To test whether P62 and BNIP3L are direct transcriptional targets of RUNX1, we performed chromatin immunoprecipitation (ChIP) assays. The results revealed that RUNX1 bound to upstream sequences proximate to the transcription start site (−1500-0 bp) of both *P62* and *BNIP3L* (Fig. 1i). To test whether the promotion of mitophagy by RUNX1 depends on the expression of *P62* or *BNIP3L* in A549 cells after CCCP treatment, we performed rescue experiments. Immunofluorescence imaging showed that RUNX1-dependent activation of mitochondrial engulfment was significantly blocked by either P62 silencing or BNIP3L silencing (Fig. 1j and Supplementary Fig. S2a–d). These data demonstrate that RUNX1 promotes mitophagy through transcriptional activation of P62 and BNIP3L expression.

Alveolar type II cells (AT2) are mitochondria-rich epithelial cells responsible for lung injury repair.^4^ To test the role of RUNX1 on mitophagy in AT2 in vivo, we induced acute lung injury in wild-type mice by intranasal administration of lipopolysaccharide (LPS) and characterized the mitophagy activation and RUNX1 expression by immunofluorescence co-staining of pro-SP-C (a marker of AT2 cells) with PARKIN (a marker of damaged mitochondria), with LC3B (a marker of autophagosome) or with RUNX1 (Supplementary Fig. S3a–c). Two hours after LPS administration, increased cytoplasmic condensation of PARKIN, LC3B, and increased nuclear staining of RUNX1 were observed in pro-SP-C positive AT2, indicating activation of mitophagy and RUNX1 expression in AT2. Twenty-four hours after LPS administration, staining of LC3B, pro-SP-C, and RUNX1 was decreased (Supplementary Fig. S3d–g). Consistent with these findings, damaged mitochondria identified by the increased gray value in AT2 were readily observed by electron microscopy 2 h after LPS injury and were significantly eliminated 24 h after LPS administration (Supplementary Fig. S3h). Thus, expression of RUNX1 is temporally associated with mitophagy activation in AT2 during LPS-induced acute lung injury.

To test whether RUNX1-dependent activation of mitophagy in AT2 was sufficient to protect the lung from LPS injury, a transgenic mouse (IE-RUNX1) in which RUNX1 conditionally expressed in AT2 was produced (Supplementary Fig. S4a). In the lungs of IE-RUNX1 mice after LPS administration, increased expression of RUNX1, P62, and BNIP3L was observed (Fig. 1k and Supplementary Fig. S4b). AT2 damage induced by LPS injury was alleviated, including rescued expression of pro-SP-C (Supplementary Fig. S4c, d), decreased cytoplasmic condensation of PARKIN (Fig. 1l and Supplementary Fig. S4e), decreased mitochondrial swelling and cristae damage (Fig. 1m). Damaged mitochondria are known to activate cGAS/STING and NLRP3 signaling to influence cytokine production. Consistent with our findings that alleviated mitochondria damage in the lungs of RUNX1 IE mice, the protein levels of cGAS, STING, and NLRP3 were significantly decreased (Fig. 1n and Supplementary Fig. S4f). The number of inflammatory cells was significantly decreased in BALF of IE-RUNX1 mice after LPS injury (Fig. 1o), associated with decreased proinflammatory cytokines, including IFN-α, IFN-β, IL-1β, IL-18, IL-6, and TNF-α (Fig. 1p–v). These data support the conclusion that RUNX1-dependent activation of mitophagy in AT2 protected the lung from LPS injury.

To test whether RUNX1-dependent mitophagy was dysregulated in human ARDS, we collected five autopsy samples from ARDS patients, including three patients that died from SARS-CoV-2. Para-cancerous normal lung tissues were used for controls. Cytoplasmic condensation of PARKIN was increased in alveolar epithelial cells of the ARDS patients, indicating inefficient clearance of damaged mitochondria by mitophagy. RUNX1 and BNIP3L staining was decreased in alveolar epithelial cells of the ARDS patients (Fig. 1w, x), and decreased staining of P62 was identified in two of the three ARDS samples (Fig. 1y). Taken together, our data support the conclusion that dysregulation of RUNX1-dependent mitophagy in AT2 cells participate in the pathogenesis of ARDS.

In summary, our findings provided evidence supporting the potential therapeutic targeting of RUNX1-dependent mitophagy to alleviate AT2 damage and pulmonary inflammation in ARDS. Additionally, there is increasing evidence indicating that SARS-CoV-2 exploits mitophagy to enhance its survival.^5^ Therefore, understanding the role of RUNX1 in mitophagy also could offer valuable insights into ARDS associated with SARS-CoV-2 infection.

## Supplementary information

Supplementary Materials

## Data Availability

All data and materials presented in the manuscript are available on request. The RNA sequence data has been deposited in theNational Center for Biotechnology Information (PRJNA947781).

## References

[1] Beitler JR (2021). Advancing precision medicine for acute respiratory distress syndrome. Lancet Respir. Med.

[2] Islam MN (2012). Mitochondrial transfer from bone-marrow-derived stromal cells to pulmonary alveoli protects against acute lung injury. Nat. Med..

[3] Palikaras K, Lionaki E, Tavernarakis N (2018). Mechanisms of mitophagy in cellular homeostasis, physiology and pathology. Nat. Cell Biol..

[4] Mokra D (2020). Acute lung injury—from pathophysiology to treatment. Physiol. Res..

[5] Singh KK, Chaubey G, Chen JY, Suravajhala P (2020). Decoding SARS-CoV-2 hijacking of host mitochondria in COVID-19 pathogenesis. Am. J. Physiol. Cell Physiol..

