# Supplementary material for: RUNX1 promotes mitophagy and alleviates pulmonary inflammation during acute lung injury
Source: Signal Transduct Target Ther. 2023 Aug 7;8:288. doi: 10.1038/s41392-023-01520-6 (PMC10404583; doi:10.1038/s41392-023-01520-6)
Supplement: Supplementary file 1 — Supplementary Materials [file 41392_2023_1520_MOESM1_ESM.docx]

Supplementary Materials for

**RUNX1 promotes mitophagy and alleviates pulmonary inflammation during acute lung injury**

Xiaoju Tang^1,2^, Lichun Zhong^2^, Xin Tian^2^, Ying Zou^2^, Silu Hu^2^, Jia Liu^1,2^, Ping Li^1,2^, Min Zhu^1,2^, Fengming Luo^1,2*^, Huajing Wan^1,2*^

*Correspondence:

Fengming Luo, E-mail: fengmingluo@outlook.com,

Huajing Wan, E-mail: wanhuajing1974@wchscu.cn

**This PDF file includes:**

Materials and Methods

Figures. S1-S4

Tables. S1-S5

**Materials and Methods**

**Human samples**

The study protocol complies with the principles of the Declaration of Helsinki. Human lung tissue was obtained at either autopsy or lobectomy under protocols approved by the Committee on Human Research West China Hospital of Sichuan University (No.2020-126, 2021-1271), including 5 autopsies from ARDS patients and 3 para-cancerous normal lung tissues obtained at lobectomy from lung cancer patients.

**Transgenic mice and animal husbandry**

Animals were maintained in pathogen-free conditions according to protocols approved by the Institutional Animal Care and Use Committee at West China University Hospital. Full-length mouse RUNX1 coding sequence was isolated from pcDNA3.1-*Runx1* (Addgene, Cat. No. 14585 ) and cloned into the ptetO_7_-IRES-EGFP vector. Transgenic mice (*tetO_7_-Runx1-IRES-EGFP^wt/tg^*) were constructed by Cyagen Biosciences (China) and were identified by PCR using the primer set: 5’-CTGCACCAACGCATCCACCC-3’ and 5’-ACACCGGCCTTATTCCAAGC-3’. For lung epithelial-specific, doxycycline-induced RUNX1 expression, the heterozygous offspring were mated with FVB. Cg^-/Tg^(*Sftpc-rtTA*^)^5Jaw/J transgenic mice ( The Jackson Laboratory, Bar Harbor, Maine) to generated *sftpc-rtTA*^-/tg^; tetO7-RUNX1^-/tg^ mice*.* These double transgenic mice termed IE-RUNX1 after exposure to doxycycline. And single transgenic mice *sftpc-rtTA*^-/tg^ or tetO7-RUNX1^-/tg^ were used as controls. All animals were housed in humidity- and temperature-controlled rooms on a 12:12 hour light:dark cycle and were allowed food and water ad libitum.

**Pulmonary LPS administration**

Adult C57BL/6N mice (6-8 weeks old) mice were obtained from the GemPharmatech (Chengdu, China). Mice under anesthesia were exposed to LPS (6 mg/kg, Escherichia coli O55:B5; Sigma) by intranasal administration. Expression of RUNX1 in the *sftpc-rtTA*^-/tg^; tetO7-RUNX1^-/tg^ mice was induced at 6-8 weeks of age by the provision of doxycycline (Sigma, USA) 5 days before mice were intranasally administrated LPS. The control mice were the single transgenic littermates that received the same doxycycline and LPS treatments. Doxycycline was continued until the time of sacrifice. Lung tissues were harvested at different time points. All experiments shown were representative of findings from at least 3 independent experiments.

**Cell culture and treatments**

A549 cells (a human lung carcinoma cell line) and MLE-12 ( a murine alveolar type II epithelial cells) were grown to 70% confluence in 12-well plates, starved for 4 hours in serum-free culture medium, then treated with 20 uM carbonyl cyanide m-chlorophenyl hydrazine (CCCP, Sigma Aldrich, USA) and harvested for analysis at different time points after CCCP treatment. Plasmids and siRNAs were transfected into cultured epithelial cells using Lipofectamine RNAiMAX (Invitrogen, USA) following the manufacturer’s instructions. Sequences of siRNAs (obtained from Ribobio) are listed in Table. S2. At least 24 hours after transfection, cells were harvested for analysis. Adenovirus encoding human RUNX1 (Adv-R) and the control adenovirus (Adv-C) were purchased from WZ Biosciences (Shandong, China), human PARKIN (Adv-Parkin), GFP-labeled-LC3 (Adv-GFP-LC3) and Adv-HBmTur-Mito (Adv-Mito-Red) were purchased from Hanbio (Shanghai, China). 36 hours after transfection, cells were harvested for analysis.

**Morphological analysis**

Lung tissue was harvested and immersion-fixed in 4% paraformaldehyde overnight. Frozen and paraffin sections were prepared and stained as previously described(Tang *et al.*, 2017). Primary antibodies used for immunofluorescence and immunohistochemistry staining were listed in Table. S3. Fluorochrome-conjugated (Alexa Fluor 488 and 550, Invitrogen) secondary antibodies were used for detection. Immunohistochemistry staining images were captured with a Nikon DS-Ri2 microscope. Immunofluorescence staining images were scanned with a Leica Stellaris 8 confocal microscope under a 63X oil objective lens. The immunofluorescence intensity of PARKIN or LC3B in pro-SPC positive cells was quantified using Imaris 9.7 software. Intensity data from more than 600 pro-SPC positive cells/mouse, 4 mice/experimental group were quantified and compared. For ultrastructural analysis, mice lung tissues and A549 cells were fixed in 3% glutaraldehyde or 0.3% glutaraldehyde in 2% paraformaldehyde and 0.1M sodium cacodylate buffer at pH7.3, respectively and embedded as previously described. And electron microscopy (JEP-1400PLUS) was performed.

**Protein, RNA and cytokine quantification**

Total proteins were prepared, and Western blot was performed. Antibodies were listed in Table. S3. RNAs were isolated by using TRIzol (Invitrogen, USA), and reverse transcribed by using Rever Tra Ace qPCR RT Kit (TOYOBO, Japan). The cDNAs were quantified by RT-qPCR and Primers were listed in Table. S4. Independently repeated experiments were performed, densitometry quantification of the Western blot bands and PCR products was carried out using Quality One Software (Bio-Rad Laboratories, Philadelphia, PA, USA), and data were expressed as the mean±SEM. To determine cytokine levels, BALF was collected as previously described(Tang *et al.*, 2017). IFN-α ELISA kit (Cat. No. RDR-IFNα-Mu, Reddot Biotech, USA), IFN-β ELISA kit (Cat. No. RDR-IFNβ-Mu, Reddot Biotech, USA) and the mouse LEGENDplexTM Multi-Analyte Flow Assay Kit (Cat. No.750000417, BioLegend, USA) were used to determine the concentrations of cytokines.

**RNA-Sequence and bioinformatic analysis**

Sequencing libraries were generated using NEBNext UltraTM RNA Library Prep Kit for Illumina (NEB, USA) following the manufacturer’s recommendations, and index codes were added to attribute sequences to each sample. The clustering of the index-coded samples was performed on a cBot Cluster Generation System using TruSeq PE Cluster Kit v3-cBot-HS (NEB, USA) according to the manufacturer’s instructions. After cluster generation, the library preparations were sequenced on an Illumina Novaseq platform and 150 bp paired-end reads were generated. Then, low-quality reads (with ≥10% unidentified nucleotides, >10 nt aligned to the adapter with ≤10% mismatches allowed, and with >50% of bases with phred quality < 5) were removed to avoid artificial bias. Furtherly, those cleaned reads were aligned to Homo Genome (GRCh38.p13, download from ENSEMBL database) by Hisat2(Kim *et al*, 2015), and htseq-count was used to extract the count matrix. Finally, Differential expression analysis was performed with the DESeq2 R package (1.16.1)(Love *et al*, 2014). The resulting p-values were adjusted using Benjamini and Hochberg’s approach for controlling the false discovery rate. Genes were considered significant for up-regulation/down-regulation if *p*<0.05 and |foldchange| >1. KEGG enrichment analysis of differentially expressed genes was implemented by the clusterProfiler R package, and the top 5 pathways were shown.

**Chromatin immunoprecipitation (ChIP) assay**

A549 cells were transfected with human Adv-RUNX1-FLAG or Adv-Control. Forty-eight hours after transfection, cells were harvested and ChIP assays were performed using SimpleChIP Plus Sonication Chromatin IP kit (Cell signaling Technology, Cat. No.56383). Rabbit IgG, anti-FLAG antibody (Cell signaling Technology, Cat. No. 14793S) and protein G agarose beads（Cell signaling Technology, Cat. No. 2729）were used to precipitate protein/DNA complexes. Precipitated DNA was analyzed by PCR with P62, BNIP3L promoter-specific primers (Table. S5) that were designed around the RUNX1 binding sites predicted by the JASPAR database. Rabbit IgG ChIP was used as the negative control. PCR products were assayed with agarose gel. Independently repeated experiments were performed (n=3), densitometry of the bands was measured using Quality One software (Bio-Rad Laboratories), and data were expressed as the mean±SEM.

**Mitochondrial engulfment assay**

Adv-GFP-LC3 and Adv-HBmTur-Mito were transfected into cells to visualize autophagosome and mitochondria respectively. Cells were fixed with 4% paraformaldehyde in PBS (pH7.4) for 30 minutes, washed with PBS 3 times, and then images were acquired with a Leica Stellaris 8 confocal microscope under a 100X oil objective lens. 10 to 24 cells were randomly selected from each experimental group, and numbers of Mito-Red positive mitochondria circled with GFP-LC3 rings per cell were manually counted in Image J (NIH). Only clearly defined mitochondrially localized rings were counted (Moore & Holzbaur, 2016a). Given that P62-mediated mitophagy is reported to be Parkin-dependent(Geisler *et al*, 2010), co-transfection of Adv-Parkin with Adv-GFP-LC3 and Adv-HBmTur-Mito was carried out in the P62 rescue experiment.

**Statistical analysis**

Values for all quantifications, including western blot, RT-qPCR, morphological analysis, and image analysis, are represented as mean ± SEM. Student’s t-test was used to determine differences between groups. Two-way ANOVA was used to determine the differences between the three groups. *p* values for significance were set to 0.05.

**Study approval**

All mouse studies were approved by the IACUC of West China Hospital of Sichuan University（no.20211149A） and adhered to NIH guidelines for the use of experimental animals. Studies on human lung tissue were approved by the Committee on Human Research West China Hospital of Sichuan University.

**SUPPLEMENTAL FIGURES**

**Figure S1**

**
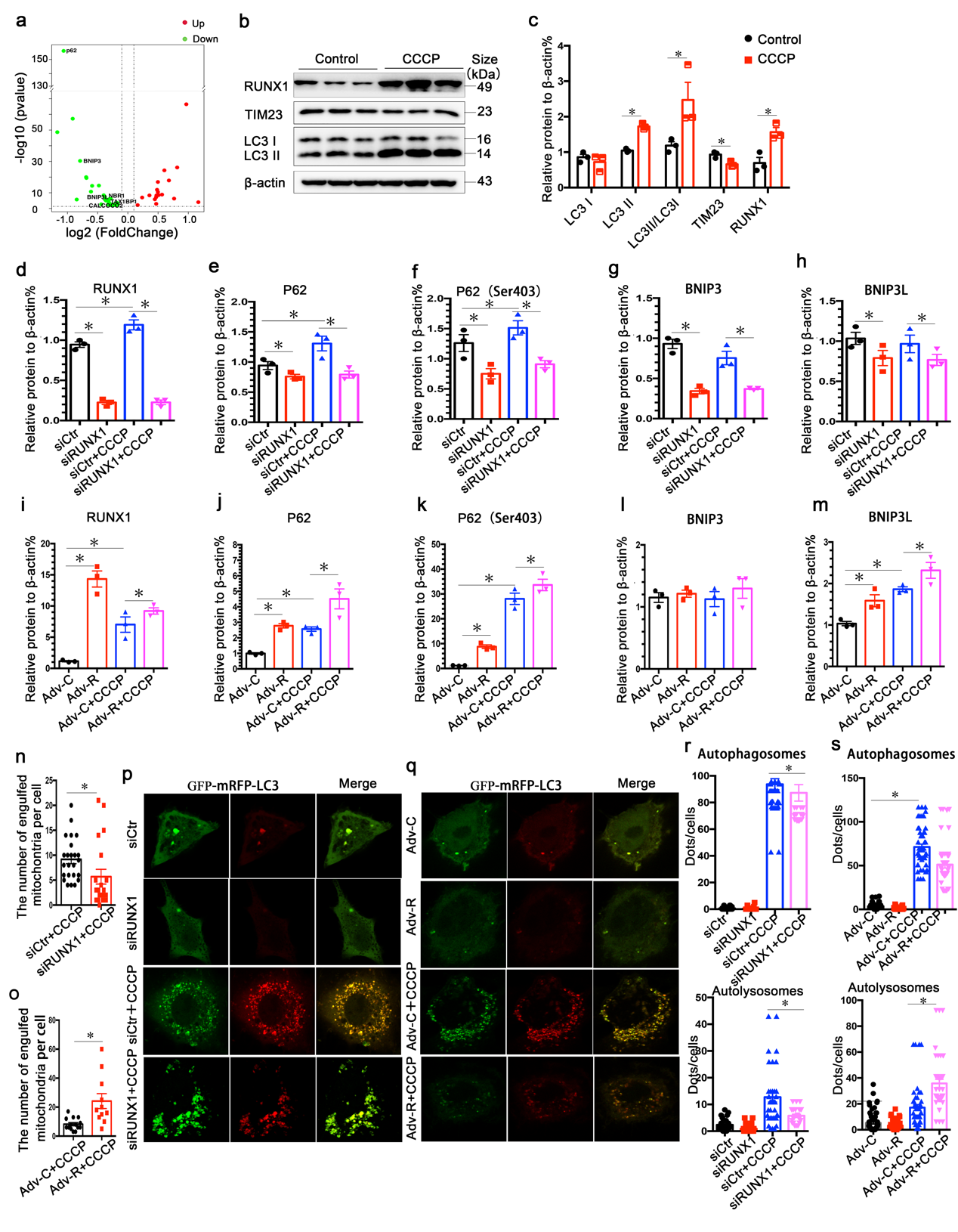
**

**Figure S1. RUNX1 promotes the engulfment of damaged mitochondria by autophagosomes. (a)** A volcano plot demonstrated that the expression of 6 mitophagy adaptor proteins was significantly decreased in RUNX1 siRNA transfected A549 cells. **(b)** Western blot for RUNX1, TIM23, LC3, and β-actin. **(c)** Quantification of western blot. Data are represented as mean±SEM (n=3 per group). (**d-m)** Quantification of western blot. Data are represented as mean±SEM (n=3 per group). **(n-o)** Quantification of autophagosomes engulfed mitochondria. Data are represented as mean±SEM (n=3 per group). (**p-q)** Representative confocal images show autophagic influx: autophagosomes (yellow puncta which merged fluorescence from RFP and GFP) and autolysosomes (free red puncta). (**r-s)** Quantification of autophagosomes and autolysosomes by Imaris 9.7 software; Data are represented as the mean ± SEM (n = 3 per group). Significance: ^*^*P* < 0.05.

**Figure S2**

**
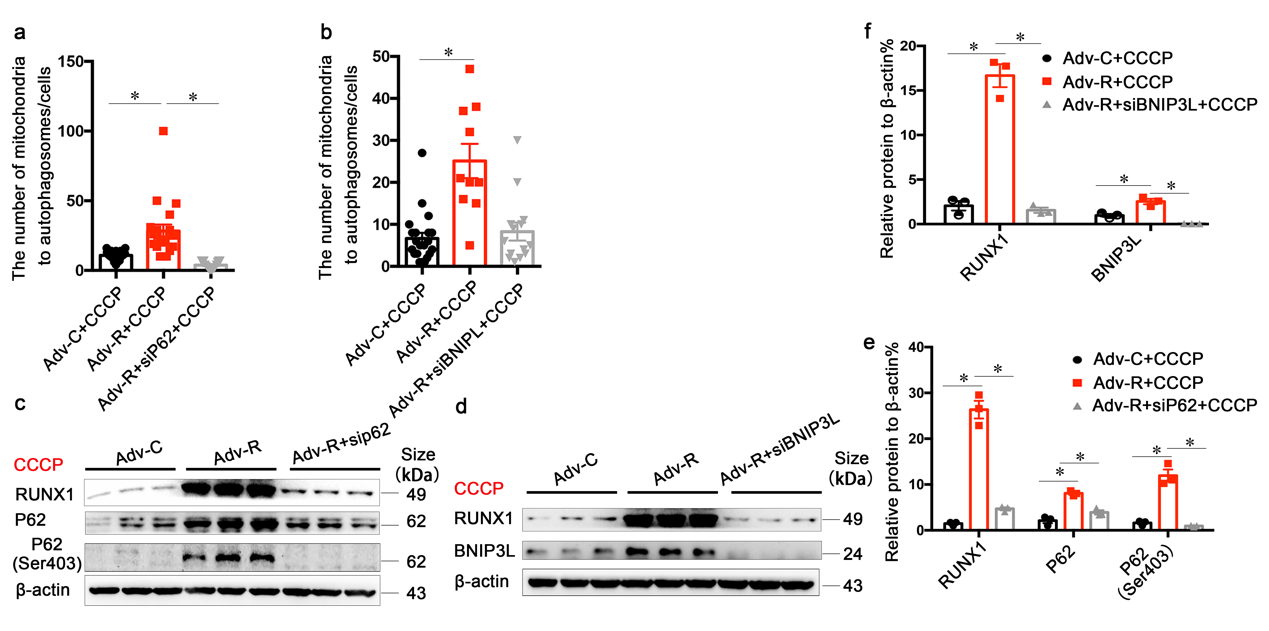
**

**Figure S2. BNIP3L or P62 silencing disrupted RUNX1 induced engulfment of damaged mitochondria by autophagosome***.* **(a-b)** Quantification of autophagosome engulfed mitochondria per cell was counted. Data are represented as mean±SEM (n=3 per group). **(c-d)** Western blot for RUNX1, P62, phosphor-P62 (Ser403), BNIP3L and β-actin. **(e-f)** Quantification of western blot. Data are represented as the mean ± SEM (n = 3 per group). ^*^*P* < 0.05.

**Figure S3**

**
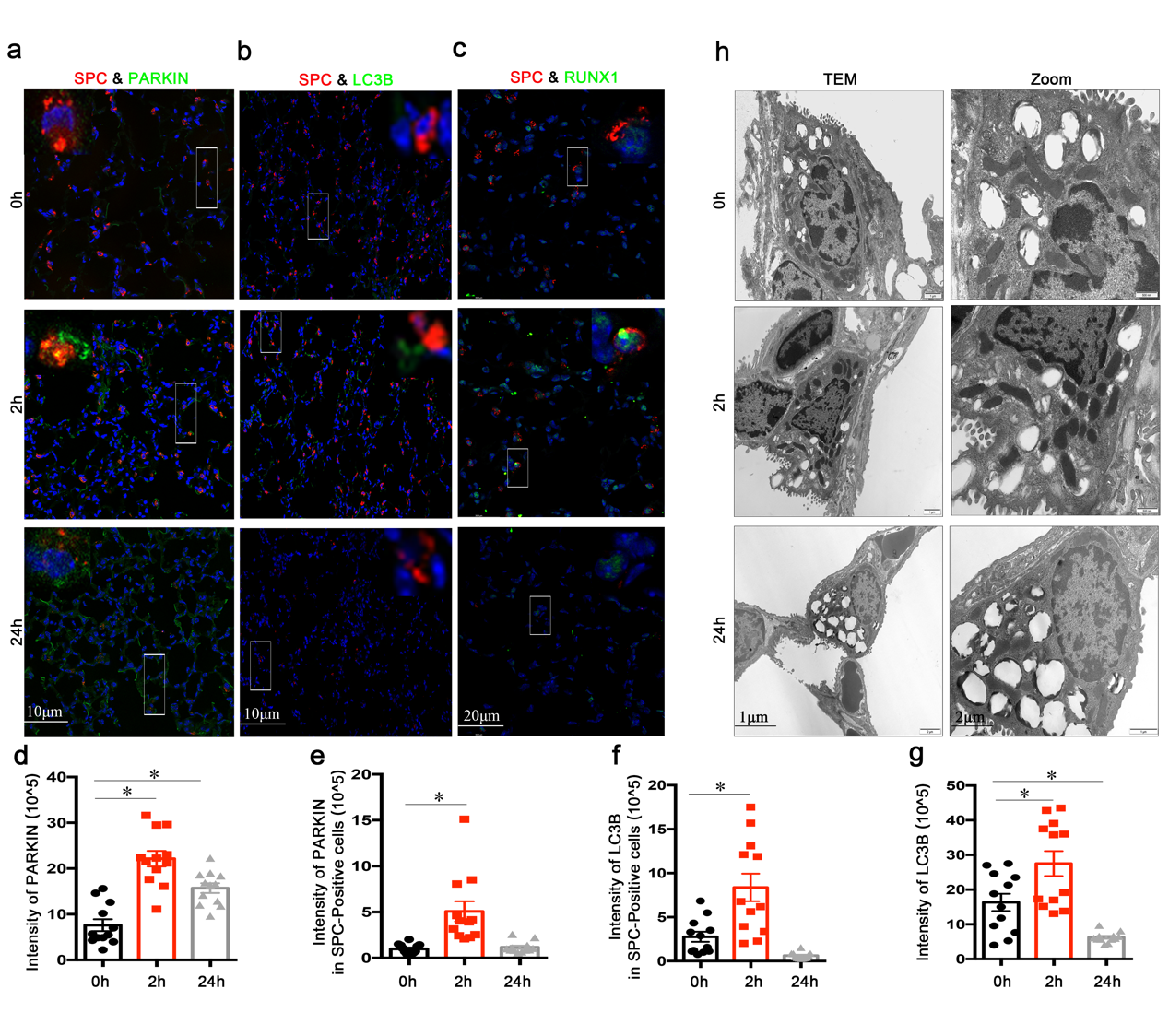
**

**Figure S3. LPS induces mitophagy and RUNX1 expression.** **(a)** Representative immunofluorescence co-staining of pro-SPC (AT2 cells marker, red) and PARKIN (damaged mitophagy marker, green), **(b)** co-staining of pro-SPC and LC3B**, (c)** co-staining of pro-SPC and RUNX1. Higher magnifications of the boxed regions are shown on the right side of the imaging. Scale bars:10μm. **(d-e)** Quantification of PARKIN intensity and LC3B intensity **(f-g)**. Data are represented as mean±SEM (n=4 per group). **(h)** Representative ultrastructure images show dynamic changes of mitochondria in AT2 cells of the lung after LPS injury. Scale bars:2μm. ^*^*P* < 0.05.

**Figure S4**

**
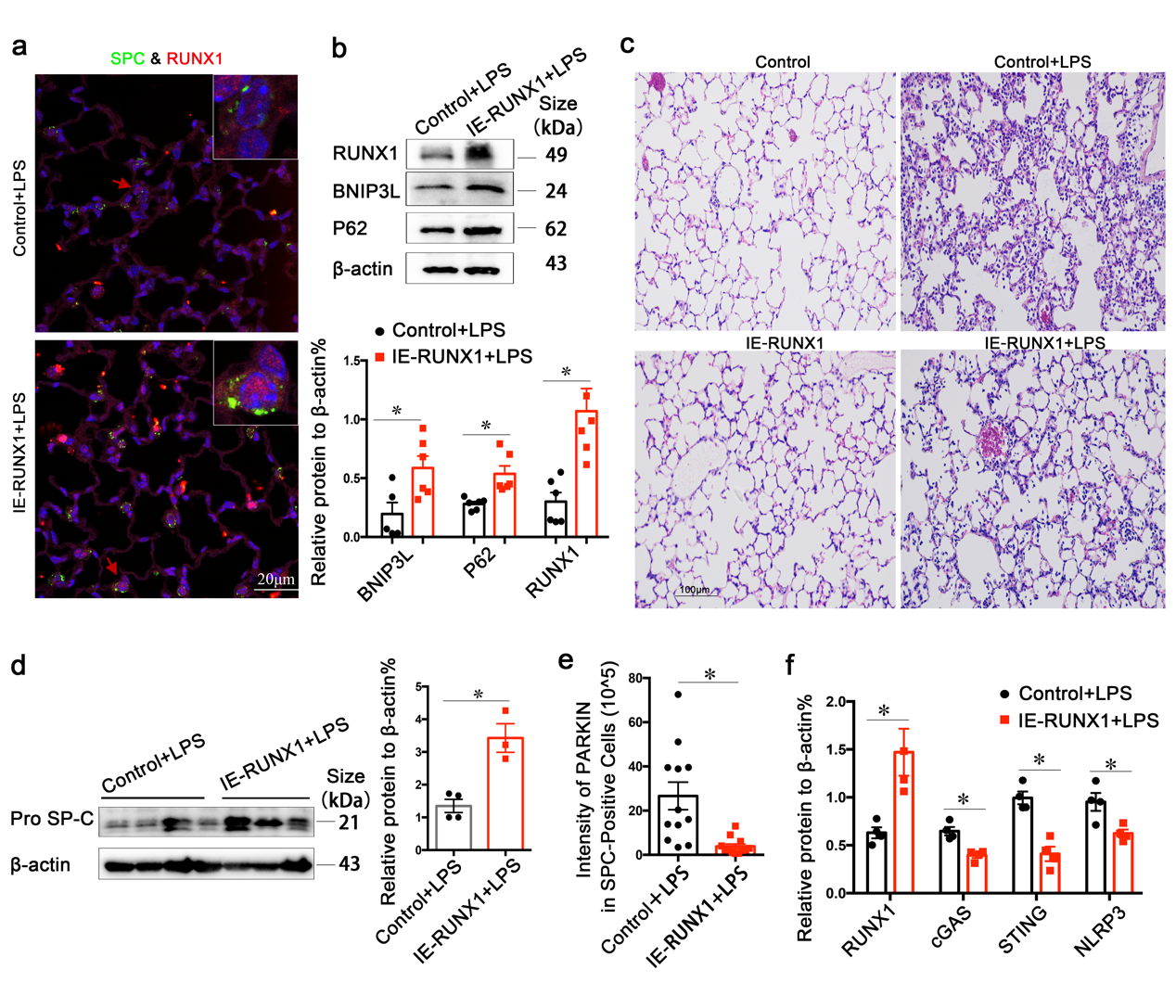
**

**Figure S4. Induced expression of RUNX1 in AT2 cells rescued LPS induced mitochondrial damage and pulmonary inflammation**. **(a)** Immunofluorescence co-staining of pro-SPC (AT2 cells marker, Green) and RUNX1 (Red). Scale bars:20μm. **(b)** Western blot of P62, RUNX1, and BNIP3L and quantification. Data are represented as mean±SEM (Control+LPS n=5, IE-RUNX1+LPS n=6). **(c)** HE staining of lung tissues from IE-RUNX1 mice (6-8 weeks) with or without LPS administration. Scale bars:100μm. **(d)** Western blot of pro SP-C and quantification. Data are represented as mean±SEM (Control+LPS n=4, IE-RUNX1+LPS n=3). **(e)** Quantification of PARKIN intensity in lung tissue of each mouse. Scale bars:10μm. Data are represented as mean±SEM (n=3 per group). **(f)** Quantification of western blot. Data are represented as mean ± SEM (n = 4 per group). ^*^*P* < 0.05.

**Table S1:** All 45 differentially expressed transcripts participating in the mitophagy pathway identified in the RUNX1 silenced A549 cells.

| **Gene_ID** | **siRUNX1** | **siCtr** | **FoldChang** | **pvalue** |
| --- | --- | --- | --- | --- |
| ENSG00000183735 | 582.9088682 | 1308.655918 | -2.245695182 | 2.16E-49 |
| ENSG00000161011 | 11646.42348 | 24329.66554 | -2.089115355 | 3.29E-156 |
| ENSG00000100644 | 5744.805406 | 10812.83806 | -1.882135875 | 5.83E-58 |
| ENSG00000254598 | 111.9464643 | 200.9568939 | -1.79765214 | 1.99E-06 |
| ENSG00000176171 | 822.422132 | 1421.604884 | -1.728897414 | 4.65E-31 |
| ENSG00000138592 | 1393.626828 | 2248.0488 | -1.612939266 | 1.04E-20 |
| ENSG00000133818 | 734.060093 | 1180.92193 | -1.608319505 | 3.91E-20 |
| ENSG00000187098 | 666.0000007 | 1017.314977 | -1.526498443 | 1.85E-11 |
| ENSG00000213281 | 1080.883296 | 1629.590547 | -1.506640278 | 3.67E-15 |
| ENSG00000185591 | 1668.635177 | 2325.563911 | -1.393870352 | 2.51E-15 |
| ENSG00000135093 | 189.0214087 | 256.6099324 | -1.356916556 | 0.002283033 |
| ENSG00000050748 | 1306.971911 | 1724.943417 | -1.319437066 | 3.64E-09 |
| ENSG00000104765 | 1093.524961 | 1421.86957 | -1.300770048 | 6.21E-08 |
| ENSG00000121749 | 718.3415071 | 932.6595518 | -1.297730358 | 2.39E-06 |
| ENSG00000135655 | 957.4058909 | 1231.596627 | -1.285167609 | 9.69E-06 |
| ENSG00000172071 | 403.8348333 | 506.4415353 | -1.253094596 | 0.00308087 |
| ENSG00000126858 | 775.8829279 | 967.7137331 | -1.246394113 | 6.65E-05 |
| ENSG00000173039 | 1644.472752 | 2041.831227 | -1.242121839 | 1.41E-06 |
| ENSG00000139112 | 626.5396182 | 766.7706987 | -1.224909348 | 0.001403838 |
| ENSG00000118689 | 397.3536021 | 485.3143156 | -1.222270891 | 0.009618448 |
| ENSG00000188554 | 1925.634195 | 2344.16094 | -1.217477042 | 6.41E-07 |
| ENSG00000057663 | 557.7923755 | 657.6970137 | -1.17955155 | 0.010398638 |
| ENSG00000133703 | 1208.9863 | 1413.240722 | -1.169194885 | 0.001952613 |
| ENSG00000101266 | 3834.24422 | 4334.817204 | -1.130565337 | 0.000247733 |
| ENSG00000136436 | 986.0207301 | 1114.134841 | -1.129359441 | 0.0147933 |
| ENSG00000106052 | 2425.401036 | 2721.96568 | -1.12208899 | 0.001406759 |
| ENSG00000170315 | 5966.320687 | 5332.185993 | 1.118900089 | 0.007033297 |
| ENSG00000197122 | 4778.501343 | 4055.523666 | 1.178249766 | 1.04E-07 |
| ENSG00000070770 | 1841.26048 | 1436.929482 | 1.280911775 | 4.61E-09 |
| ENSG00000196683 | 744.738785 | 546.8576644 | 1.362748088 | 1.48E-06 |
| ENSG00000214253 | 713.1361199 | 515.6761347 | 1.382976347 | 6.38E-08 |
| ENSG00000158828 | 291.0601908 | 210.4902925 | 1.384118065 | 0.00109341 |
| ENSG00000126581 | 966.3725937 | 695.5868661 | 1.388633806 | 4.53E-09 |
| ENSG00000177169 | 2769.481292 | 1980.213323 | 1.397803334 | 1.18E-12 |
| ENSG00000177606 | 1521.694446 | 1078.16023 | 1.411153372 | 3.08E-11 |
| ENSG00000141510 | 2509.014206 | 1777.941476 | 1.411367059 | 1.28E-17 |
| ENSG00000171109 | 778.7118624 | 547.6914206 | 1.422546646 | 3.11E-09 |
| ENSG00000174775 | 1180.604727 | 797.7083116 | 1.47964648 | 1.46E-09 |
| ENSG00000247077 | 2101.38503 | 1382.128064 | 1.520168032 | 4.98E-25 |
| ENSG00000126458 | 1312.090346 | 819.0630383 | 1.601328229 | 1.41E-18 |
| ENSG00000158186 | 245.6769851 | 144.5470441 | 1.699507961 | 5.16E-07 |
| ENSG00000101412 | 1441.197029 | 817.8848276 | 1.760768496 | 7.22E-27 |
| ENSG00000171552 | 3791.509854 | 1938.557209 | 1.955019575 | 3.55E-67 |
| ENSG00000112561 | 70.92054803 | 31.53695223 | 2.244275818 | 9.72E-05 |

**Table S2 Sequences of siRNA.** In the silencing experiment, we used at least two different sequences, and the knockout efficiency and results were consistent between different sequences. This study used human RUNX1-1, human P62-1, human BNIP3L-1, and mouse RUNX1-1 sequences for experiments.

| **Species** | **Gene Name** | **Sequence (5'-3')** | **Product Size （bp）** |
| --- | --- | --- | --- |
| Human | *P62*-1 | GGAGTCGGATAACTGTTCA | 19 |
| Human | *P62*-2 | TGAGGAAGATCGCCTTGGA | 19 |
| Human | *BNIP3L*-1 | GAATCAGGACAGAGTAGTT | 19 |
| Human | *BNIP3L*-2 | GGAAAAGTGGAGCCATGAA | 19 |
| Human | *RUNX1*-1 | GCACTCTGGTCACTGTGAT | 19 |
| Human | *RUNX1*-2 | CCAGGTTGCAAGATTTAAT | 19 |
| Mouse | *RUNX1*-1 | GCAGAACTGAGAAATGCTA | 19 |
| Mouse | *RUNX1*-2 | CCTCCTACCATCTATACTA | 19 |

**Table S3. Primary antibodies**

| **Name** | **Company** | **Cat No.** | **Concentration** | **Assays** |
| --- | --- | --- | --- | --- |
| SPC | Abcam | ab211326 | 1：1000 | IF |
| Parkin | Santa | sc-32282AF488 | 1：100 | IF |
| LC3B | Cell signaling Technology | 3868 | 1：500 | IF |
| LC3 | ProteinTech | 14600-1P | 1:1000 | WB |
| RUNX1 | Lifespan | LS-B14769 | 1：100/1:1000 | IHC/WB |
| BNIP3L | Novus | NBP1-88558 | 1：100/1:1000 | IHC/WB |
| BNIP3 | Cell signaling Technology | 44060 | 1:1000 | WB |
| P62 | HuaBio | R1309-8 | 1:100 | IHC |
| P62 | Abcam | 8052 | 1:1000 | WB |
| P62(Ser403) | Cell signaling Technology | 39786 | 1:1000 | WB |
| TIM23 | BD | 611222 | 1:1000 | WB |
| β-actin | Absin | 100041 | 1:5000 | WB |

**Table S4. Primers for RT-qPCR**

| **Species** | **Gene Name** |  | **Sequence (5'-3')** | **Product Size （bp）** |
| --- | --- | --- | --- | --- |
| Human | *RUNX1* | Forward | GTGGTCCTACGATCAGTCCT | 116 |
|  |  | Reverse | GTTCTGCAGAGAGGGTTGTC |  |
| Human | *P62* | Forward | GATGGGAAATGGGTCCACCA | 178 |
|  |  | Reverse | TCAACTTCAATGCCCAGAGGG |  |
| Human | *BNIP3L* | Forward | GAATCAGGACAGAGTAGTTCC | 150 |
|  |  | Reverse | TTCCTTCTCTCCTTCTACAAC |  |
| Human | *BNIP3* | Forward | AGAGCTTCTGAAACAGATACC | 175 |
|  |  | Reverse | GTTTAAAGAGGAACTCCTTGG |  |
| Human | *GAPDH* | Forward | CCAAAAGGGTCATCATCTCT | 90 |
|  |  | Reverse | TCTTGAGGCTGTTGTCATAC |  |
| Mouse | *RUNX1* | Forward | GATAATGGAGCTGTTGGGAA | 147 |
|  |  | Reverse | AAGGGCTATTGGGAGGATAT |  |
| Mouse | *P62* | Forward | GGACCCATCTACAGAGGCTG | 165 |
|  |  | Reverse | GGTGGAGGGTGCTTCGAATA |  |
| Mouse | *β-actin* | Forward | GTGACGTTGACATCCGTAAAGA | 245 |
|  |  | Reverse | GCCGGACTCATCGTACTCC |  |

**Table S5. Primers for ChIP-PCR**

| **Species** | **Gene Name** |  | **Sequence (5'-3')** | **Product Size （bp）** |
| --- | --- | --- | --- | --- |
| Human | *P62* | Forward | ACCTGGTACTACTGGGTCAC | 178 |
|  |  | Reverse | AGGATCCTGTGAGGTATGAG |  |
| Human | *BNIP3L* | Forward | GTATTGCAGATAGCCCTGAA | 90 |
|  |  | Reverse | AGCCATCCTTTCTACACTGA |  |
